# Consulting Dr. Google: Two experimental studies on seeking mental health information online

**DOI:** 10.1016/j.invent.2026.100992

**Published:** 2026-08-21

**Authors:** Katharina U. Siebenhaar, Georg W. Alpers

**Affiliations:** Department of Psychology, School of Social Sciences, University of Mannheim, Mannheim, Germany

**Keywords:** Internet search, Mental health information, eHealth literacy, Information accuracy, Health decision-making

## Abstract

The internet is a widely used source for mental health information, yet it remains unclear whether online searches lead to a more thorough understanding of mental disorders. This study examined whether individuals find accurate mental health information online and whether searching the internet enhances their sense of preparedness for health decision-making in two experiments. In Experiment 1 (*N* = 243), we randomly assigned participants to one of three case vignettes describing a close friend experiencing symptoms of a well-known mental disorder (depression), a lesser-known mental disorder (social phobia), or a non-clinical problem (grief). They were further randomly allocated to either an internet group, which identified the disorder and answered questions about its treatment by searching the internet, or a control group which relied on prior knowledge to complete the same task. Afterward, all participants rated how prepared they felt to make related health decisions. Experiment 2 (*N* = 252) followed the same procedure but asked participants to imagine experiencing the symptoms themselves and replaced the grief vignette with one about anorexia nervosa, a moderately-known mental disorder. In both experiments, internet searches improved answer accuracy for social phobia but not for depression or anorexia nervosa. Furthermore, participants did not feel better prepared for health decision-making following a web search. These findings suggest that web searches lead to an information gain - and potentially increase knowledge - about lesser-known disorders, but do not raise knowledge of better-known mental disorders or improve preparedness for health decision-making.

## Highlights

• This study combines case vignettes, commonly used in mental health literacy research, with an internet search task.
• Results show that internet searches facilitate information gain for lesser-known mental disorders.
• Individuals do not feel better prepared for health decision-making after searching the internet.
• Experiment 2 replicates the main findings of experiment 1.

## 1 Introduction

Recognizing a mental health problem is a crucial first step toward seeking appropriate care. However, this recognition often presents a considerable challenge, as evidenced by the variable – and frequently low – recognition rates of different mental disorders in the general population (Reavley and Jorm, 2011a). Vignette studies, the predominant methodology in mental health literacy research, indicate that 50–74% of individuals are able to correctly identify depression (Reavley and Jorm, 2011a; Sai and Furnham, 2013; Svensson and Hansson, 2016). In contrast, recognition rates for other mental disorders are substantially lower. For example, only 16–35% of individuals correctly identify anorexia nervosa (Darby et al., 2012; Nishida-Hikiji et al., 2021), and merely 7–10% recognize social phobia (Furnham and Lousley, 2013; Hadjimina and Furnham, 2017; Reavley and Jorm, 2011a). Awareness of available treatments is typically also higher for better-known disorders and lower for lesser-known disorders, such as anxiety and eating disorders (Darby et al., 2012; McNicholas et al., 2016; Reavley and Jorm, 2011a). This limited awareness represents a significant barrier to help-seeking (Gulliver et al., 2010), which may be overcome through an internet search.

Today, the internet is among the most widely used sources for mental health information (Fox and Duggan, 2013; Mahmoodi et al., 2022), with recent studies indicating that 26–52% of the general population (Bach and Wenz, 2020; Deutschland-Barometer Depression, 2025; Freytag et al., 2023; Siebenhaar et al., 2020) and up to 89% of young adults with mental health concerns, engage in online mental health information-seeking (Gowen, 2013). This trend offers substantial advantages: Online mental health resources can enhance health knowledge (Brijnath et al., 2016), foster better disease management (Bizzotto et al., 2023; Kruse et al., 2015), and empower individuals to take a more active role in their care (Pretorius et al., 2020; Sillence et al., 2007). However, the sheer volume of available content (Eysenbach and Köhler, 2002; Starcevic and Berle, 2013), combined with the generally low to moderate quality of online mental health information (Klil et al., 2013; Rathod et al., 2019; Reavley and Jorm, 2011b) can make it difficult for individuals to discern accurate and reliable content. Consequently, these limitations may reduce the potential benefits of online mental health information-seeking.

A review examining a wide range of digital information interventions suggests that such approaches are effective in increasing knowledge about lesser-known mental disorders, but not about more familiar conditions (Tay et al., 2018; Taylor-Rodgers and Batterham, 2014). However, this review takes a broad perspective, and it is unclear whether these findings specifically apply to internet searches – arguably the most prevalent method of online information-seeking. It seems plausible that internet searches are particularly effective in improving knowledge about unfamiliar disorders. This would be a great advantage as individuals frequently turn to the internet to learn about health conditions they are less familiar with (Bachofner et al., 2024). However, an internet search may also enhance the understanding of better-known mental disorders, as a certain level of familiarity could help individuals identify accurate and trustworthy information (Diviani et al., 2015). In light of this, the primary aim of the present study was to examine whether an internet search improves individuals' ability to recognize a mental disorder and find accurate treatment information, and whether it makes a difference if the disorder is lesser-known or better-known.

Through this access to information and resources, an internet search could prepare and support individuals in making informed health decisions (Chen et al., 2018; Lagan et al., 2010). In line with this, one study showed that engaging with online health information helps most individuals feel better prepared to communicate effectively with health professionals and increases their confidence in making informed decisions (Lagan et al., 2010). Given this, a web search – especially one that leads to an information gain – should enhance individuals' preparedness for mental health decision-making. Therefore, the second aim of the present study was to assess whether searching the internet makes individuals feel better prepared to make mental health-related decisions, such as pursuing treatment or taking appropriate medication.

We conducted two online experiments to test these aims. To ensure comparability with previous mental health literacy research and capture the information gain associated with internet use, both experiments combined mental health case vignettes with an instructed web search. In Experiment 1, participants read a vignette describing a close friend either experiencing symptoms of a well-known mental disorder, a lesser-known mental disorder, or a non-clinical problem, and either searched the internet or relied on their existing knowledge when answering questions about diagnosis and treatment. We hypothesized that an internet search would improve answer accuracy (accuracy hypothesis) and increase perceived preparedness for health decision-making (decision hypothesis). We also assessed whether these effects varied between lesser- and better-known mental disorders (knowledge research question). Experiment 2 constituted a conceptual replication that extended this paradigm by asking participants to imagine experiencing the symptoms themselves and by incorporating methodological refinements to strengthen the validity of the findings.

## 2 Experiment 1

### 2.1 Method

#### 2.1.1 Participants

The study was approved by the Ethics Committee of the University of Mannheim (statement 19/2021). An a priori power analysis was conducted using G*Power 3.1 (Faul et al., 2009), assuming a medium effect size (*f* = 0.25). The target sample size was 251 participants, and the final sample comprised 243 participants (*M* age = 33.16, *SD* = 13.12; 75.4% female; 53.7% with college education). These participants were recruited from the community and via social media, were required to be at least 18 years old, and provided written informed consent for participation. Exclusion criteria included: (1) studying psychology or medicine, (2) holding a degree in psychology or medicine, or (3) working in the mental health field. In addition, participants who completed the experiment unusually quickly (i.e., in less than 10 min) were excluded. Of the 243 participants, 123 were assigned to the internet group and 120 to the control group. Demographic data for each group are presented in Table 1.

**Table 1:** Demographics, mental health background, and health competences in the internet group and the control group.

A) Experiment 1 (*N* = 243)	Internet group (*n* = 123)	Control group (*n* = 120)	t/*X*^*2*^	*p*	*d*
	*M* (*SD*)/%	*M* (*SD*)/%			
Demographics					
Age	31.78 (12.58)	34.58 (13.56)	−1.67	0.096	0.214
Female gender	78	72.5	1.08	0.299	
University degree	51.3	56.7	0.84	0.359	

Mental health background					
Depression	11.4	9.2	0.32	0.361	
Anxiety disorders	4.9	7.5	0.72	0.281	
Other mental disorder	10.6	5.8	1.80	0.133	
Received mental health treatment	28.7	30.8	0.340	0.329	

Health competence					
eHealth literacy	27.64 (5.47)	27.83 (5.31)	−0.27	0.786	0.035
Mental health knowledge	25.88 (3.17)	25.22 (2.84)	1.72	0.087	0.219


B) Experiment 2 (*N* = 252) – replication	Internet group (*n* = 123)	Control group (*n* = 129)	t/*X*^*2*^	*p*	*d*
	*M* (*SD*)/%	*M* (*SD*)/%			
Demographics					
Age	29.94 (9.49)	31.58 (11.43)	−1.24	0.216	0.156
Female gender	76.4	79.1	0.26	0.651	
University degree	56.9	61.2	0.49	0.523	

Mental health background					
Depression	21.1	16.3	0.98	0.204	
Anxiety disorders	11.4	14.3	0.02	0.526	
Other mental disorders	8.9	3.9	2.72	0.082	
Received mental health treatment	35.9	28.8	1.39	0.148	

Health competence					
eHealth literacy	30.09 (6.05)	30.19 (5.09)	−0.148	0.882	0.018
Mental health knowledge	47.05 (3.62)	46.33 (4.30)	1.43	0.155	0.181

Note. eHealth literacy was measured with the eHEALS (Norman and Skinner, 2006). Mental health knowledge was assessed using different tools in Experiment 1 (the Mental Health Knowledge Schedule; MAKS; Evans-Lacko et al., 2010) and in Experiment 2 (Items 1–15 of the Mental Health Literacy Scale; MHLS; O'Connor and Casey, 2015) Mental health background refers to self-reported previous experience with depression, anxiety disorders, other mental health disorders, and previous treatment for a mental disorder.

#### 2.1.2 Procedure

Participants first provided sociodemographic information and completed self-report measures assessing mental health knowledge (Evans-Lacko et al., 2010) and eHealth literacy (Norman and Skinner, 2006). Next, participants were randomly assigned within SoSci Survey using the platform's built-in, fully automated urn randomization function, which ensured approximately balanced allocation across the two experimental groups (control, internet-search) and the three vignette conditions (depression, social phobia, or grief). The control group answered the questions based on their own knowledge, while participants in the internet group were instructed to search for answers to the questions online. Participants then completed measures of preparation for decision-making (Graham and O'Conner, 2010), and general use of online mental health information. Finally, participants were debriefed thoroughly, and they were given the option to participate in a raffle to win Amazon vouchers. No other compensation was provided.

#### 2.1.3 Materials

##### 2.1.3.1 Digital health information use

Using self-generated items, we assessed how frequently participants had previously used mental health apps or searched the web for mental health information. Furthermore, we asked participants whether they would consider online mental health information when making health decisions.

##### 2.1.3.2 Case vignettes and questions

The three case vignettes described a person (gender counterbalanced) experiencing symptoms of either depression, social phobia, or grief following the recent loss of a family member. Participants were asked seven questions related to the vignettes, which were developed and scored with respect to the German national S3 treatment guidelines for depression (Bundesärztekammer et al., 2022; DGPPN et al., 2015) and anxiety disorders (Bandelow et al., 2021). Two questions focused on identifying the disorder: (1) recognizing the symptoms as indicative of a mental disorder, and (2) correctly labelling the disorder. The remaining five questions addressed treatment-related information, including (3) whether professional help is needed, (4) which type of health professional should be consulted, (5) which treatments are most helpful, (6) what medication is appropriate, and (7) whether psychiatric hospital admission is necessary. Question (2) regarding the specific diagnosis was open-ended; all other questions were multiple choice with one or more correct answers. To ensure equal weighting, all items were scored dichotomously (0 = incorrect, 1 = correct), and a percentage score reflecting overall accuracy was calculated. The vignettes, the exact wording of the questions as presented in the experiment, and the corresponding answers are provided in the supplementary material.

##### 2.1.3.3 Preparation for decision-making

The Preparation for Decision-Making Scale (PrepDM; Graham and O'Conner, 2010) consists of 10 items assessing how prepared individuals feel to make a health decision on a 5-point Likert scale (1 = “not at all”, 5 = “a great deal”). The scale comprises two subscales: one (7 items) assesses general preparedness, while the other (3 items) evaluates feeling prepared to interact with a healthcare practitioner during the decision-making process. Previous studies have demonstrated excellent internal consistency for the PrepDM (Cronbach's α = 0.95; Buchholz et al., 2011), which was similar in the present studies (Study 1: Cronbach's α = 0.93; Study 2: Cronbach's α = 0.92). A validation study of the German version confirmed its two-factor structure and supports its use in combination with web-based decision aids (Buchholz et al., 2011).

##### 2.1.3.4 Mental health knowledge

Mental health knowledge was measured using the Mental Health Knowledge Scale (MAKS; Evans-Lacko et al., 2010). The 12-item scale includes items 1–6, which assess stigma-related knowledge on a 5-point Likert scale (1 = “strongly disagree”, 5 = “strongly agree”), with an additional opt-out option (“I don't know”) scored as 3. Items 7–12 assess the recognition of mental disorders. In our sample, the internal consistency of the scale was low, with a Cronbach's α = 0.49, compared to α = 0.65 in the original literature (Evans-Lacko et al., 2010).

##### 2.1.3.5 eHealth literacy

eHealth literacy was assessed using the eHEALs, a widely used, 8-item scale with good internal consistency (Norman and Skinner, 2006). This scale assesses an individual's self-reported ability to find and use accurate health information online. Responses were recorded on a 5-point Likert scale (1 = “strongly disagree”, 5 = “strongly agree”). Internal consistency was good in both experiments (Experiment 1: Cronbach's α = 0.89; Experiment 2: Cronbach's α = 0.89).

#### 2.1.4 Data analysis

Statistical analyses were conducted using SPSS 27 (IBM Corp., 2020). Before conducting the analysis, we verified that all assumptions were met and applied appropriate corrections where necessary and interpreted effect sizes following Cohen (Cohen, 1988). We first calculated descriptive statistics on participants' self-reported use of digital health information sources. To test our accuracy hypothesis, we first computed an index for the percentage of accurate answers across all seven questions. We then conducted a 2 × 3 ANCOVA with the percentage of accurate answers as the dependent variable and the between-subject factors group (internet group vs. control group) and disorder (depression vs. social phobia vs. grief, i.e., no disorder). Next, we tested our decision hypothesis by running a second 2 × 3 ANCOVA with preparation for decision making as the dependent variable and group (internet group vs. control group) and disorder (depression, social phobia, grief) as between-subject factors. Mental health knowledge, use of mental health apps, and prior experience with online mental health information seeking as well as eHealth literacy were explored as covariates in the model to control for potential confounding influences. In both analyses, we followed up on significant main effects and interactions. Unless otherwise specified, statistical significance was evaluated at α = 0.05. Where appropriate, significance thresholds were adjusted to account for multiple comparisons.

### 2.2 Results

#### 2.2.1 Descriptives: digital health information use

Regarding the use of mental health apps, 0.8% of participants reported using them very often (i.e., daily), 4.1% used them often, 9.1% used them sometimes, 14.8% used them rarely, and 71.2% never used them. In contrast, internet use for mental health information was more frequent: 3.3% used it very often, 12.8% used it often, 34.4% used it sometimes, 29.6% used it rarely, and 19.4% never used it. Furthermore, 88.9% of participants reported that they would use online mental health information for health decision-making.

#### 2.2.2 Accuracy hypothesis

A 2 (group: control vs. internet) × 3 (vignette: depression vs. social phobia vs. grief) ANCOVA, with percentage of accurate answers as the dependent variable and mental health knowledge, eHealth literacy, use of mental health apps, and prior online health information seeking as covariates, revealed a significant interaction effect between group and disorder, *F*(2, 233) = 6.59, *p* = 0.002, partial *η*^*2*^ = 0.054, as well as a significant main effect for disorder, *F*(2, 233) = 13.33, *p* < 0.001, partial *η*^*2*^ = 0.103. The main effect for the factor group, *F*(1, 233) = 2.47, *p* = 0.117, partial *η*^*2*^ = 0.011, and the covariates were not significant, all *F*'s ≤ 2.02, all *p's* ≥ 0.156, partial *η*^*2*^*'s* ≤ 0.009.

Follow-up tests for the interaction effect (significance threshold of α = 0.0167 [0.05/3]) revealed that the internet group (*M* = 75.96, *SD* = 21.53) answered more questions correctly about social phobia than the control group (*M* = 58.44, *SD* = 17.10), *t*(83) = 4.17, *p* < 0.001, *Cohen's d* = 0.90. However, there were no significant differences between the internet group (*M* = 82.31, *SD* = 20.16) and the control group (*M* = 81.08, *SD* = 14.69) in the percentage of accurate answers for depression, *t*(77) = 0.31, *p* = .378, *Cohen's d* = 0.07. Similarly, there were no significant differences between the internet group (*M* = 76.43, *SD* = 22.54) and the control group (*M* = 81.69, *SD* = 18.81) regarding the percentage of accurate answers for grief, *t*(77) = −1.12, *p* = 0.132, *Cohen's d* = −0.25.

To follow up on differences in the percentage of correct answers provided for the different vignettes, one-way ANOVAs were conducted for the experimental and control group separately. In the control group, differences were significant; *F*(2, 117) = 25.41, *p* < 0.001. Post hoc comparisons using Tukey's HSD test indicated that participants gave significantly fewer correct answers for social phobia compared to depression, MD = 22.64, *p* < 0.001, and grief, MD = 23.24, *p* < 0.001. In the experimental group, the percentage of correct answers was similar across all vignettes, *F*(2, 120) = 1.14, *p* = 0.325. These findings show that through an internet search, participants gain novel information about social phobia, but not about depression or grief. See Fig. 1 for an illustration of the effect and see Table 2 for the percentage of correct answers given to each question in all subgroups.

**Fig. 1:**
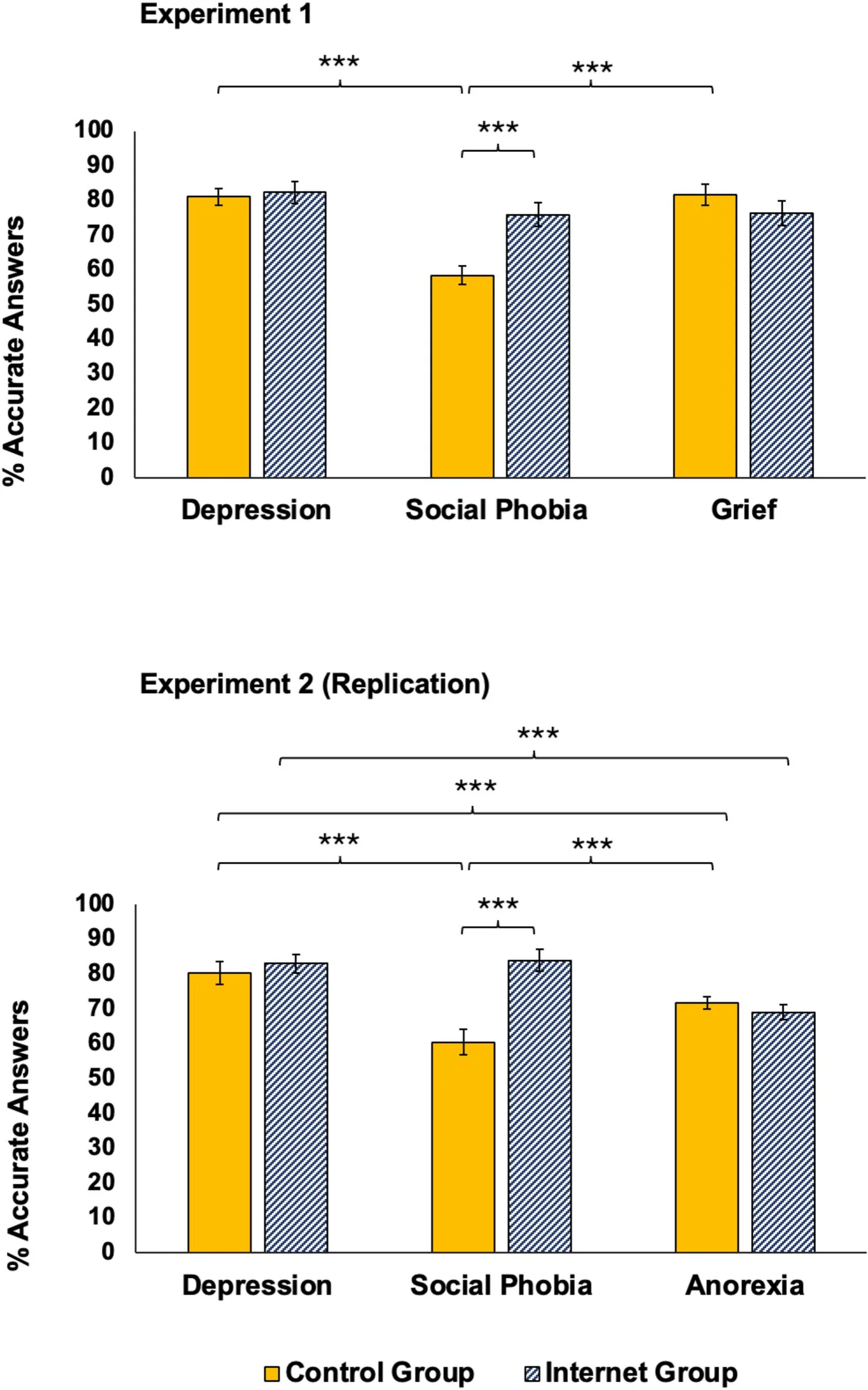
Percentage of accurate answers in the internet group and the control group. In Experiment 1 the percentage of accurate answers regarding social phobia is significantly lower in the control group, compared with the internet group (*p* < 0.001). Further, the control group gives less accurate answers about social phobia, compared with depression or grief, all *p*'s < 0.001. In Experiment 2, the lower accuracy for social phobia in the control group relative to the internet group was replicated. In addition, participants provided less accurate answers for anorexia nervosa compared with depression in the control and internet group. *** indicate *p* < 0.001.

**Table 2:** Percentage of accurate answers to questions in the internet group and the control group.

Experiment 1		Depression			Social phobia			Grief		
		Control group (*n* = 37)	Internet group (*n* = 42)		Control group (*n* = 44)	Internet group (*n* = 41)		Control group (*n* = 39)	Internet group (*n* = 40)	
		%	%	*p*	%	%	*p*	%	%	*p*
Questions:										
(1)	Recognition of a mental health problem	97.3	88.1	0.132	70.5	78	0.291	97.4	92.5	0.317
(2)	Identified the disorder	70.3	71.4	0.553	9.1	58.5	<0.001⁎⁎	100	95	0.253
(3)	Recognized that professional help is needed	97.3	95.2	0.548	93.2	97.6	0.335	87.2	85	0.518
(4)	Identified the type of health professional that should be consulted	97.3	100	0.468	88.6	95.1	0.247	66.7	62.5	0.440
(5)	Identified the most helpful treatments	78.4	71.4	0.328	54.5	75.6	0.035	56.4	52.5	0.451
(6)	Identified appropriate medication	51.4	64.3	0.157	2.3	34.1	<0.001⁎⁎	64.1	52.5	0.207
(7)	Accurate recommendation regarding admission to a psychiatric ward	75.7	85.7	0.198	90.9	92.7	0.540	100	95	0.253


Experiment 2 - replication		Depression			Social phobia			Anorexia nervosa		
		Control group (*n* = 43)	Internet group (*n* = 38)		Control group (*n* = 42)	Internet group (*n* = 42)		Control group (*n* = 44)	Internet group (*n* = 43)	
		%	%	*p*	%	%	*p*	%	%	*p*
Questions:										
(1)	Recognition of a mental health problem	90.7	97.4	0.221	76.8	92.9	0.034	100	97.7	0.494
(2)	Identified the disorder	81.4	76.4	0.386	31	73.8	<0.001⁎⁎	58.1	68.2	0.227
(3)	Recognized that professional help is needed	86	94.7	0.176	81	88.1	0.274	100	100	
(4)	Identified the type of health professional that should be consulted	97.7	97.4	0.721	83.3	97.6	0.029	100	95.3	0.241
(5)	Identified the most helpful treatments	67.4	71.1	0.457	50	83.3	0.001⁎	86.4	74.4	0.128
(6)	Identified appropriate medication	44.2	57.9	0.157	4.8	59.5	<0.001⁎⁎	11.4	14	0.483
(7)	Accurate recommendation regarding admission to a psychiatric ward	95.3	86.8	0.168	97.6	92.9	0.308	36.4	44.2	0.300

*Note.* Correct answers varied depending on the vignette. For a detailed overview of the answers and the exact wording of the questions as presented in the experiment, see the supplementary material. *p*-Values (obtained from chi-square tests) are one-sided. Statistical significance was evaluated using a Bonferroni-corrected significance threshold of α = 0.0024 (0.05/21) to account for multiple testing.

⁎ *p* < 0.01.

⁎⁎ *p* < 0.001.

#### 2.2.3 Decision hypothesis

A 2 (group: control vs. internet) × 3 (vignette: depression vs. social phobia vs. grief) ANCOVA, with preparation for decision-making as the dependent variable and the covariates mental health knowledge, eHealth literacy, use of mental health apps, and use of online mental health information, revealed no significant interaction effect for group x disorder, *F*(2, 233) = 1.94, *p* = 0.146 partial *η*^*2*^ = 0.016. Further, the main effect for group was not significant, *F*(1, 233) = 1.05, *p* = 0.307, partial *η*^*2*^ = 0.004, but the main effect for disorder was significant, *F*(2, 233) = 11.09, *p* < 0.001, partial *η*^*2*^ = 0.087. Mental health knowledge, *F*(1, 233) = 6.73, *p* < 0.001, partial *η*^*2*^ = 0.028, and use of online mental health information, *F*(1, 233) = 9.03, *p* < .001, partial *η*^*2*^ = 0.037, and eHealth literacy, *F*(1, 233) = 3.64, *p* = 0.059, partial *η2* = 0.015, were significant covariates, while the use of mental health apps was not significant, *F*(1, 233) = 0.17, *p* = 0.677, partial *η*^2^ = 0.001. Follow-up tests for the main effect (significance threshold of α = 0.025 [0.05/2]) of disorder showed that, across both groups, participants felt more prepared to make health decisions regarding depression or social phobia than regarding grief, with all *p'*s < 0.001.

### 2.3 Interim discussion

We assessed whether a web search enables individuals to gain novel information about mental disorders and subsequently feel prepared to make informed health decisions. To our knowledge, this is the first study to evaluate individuals' ability to locate mental health information online in an experimental setting. Results showed that individuals were able to find novel information about social phobia, a lesser-known mental disorder, but not about depression, a better-known disorder. This suggests that web searches are particularly helpful in informing individuals about lesser-known disorders (Tay et al., 2018). However, even when individuals gained knowledge, they did not feel better prepared to make health-related decisions. Therefore, we provide partial support for our accuracy hypothesis but refute our decision hypothesis.

The results should be considered in light of several limitations. First, the vignette described “a close friend”, and we subsequently instructed participants to search for information on behalf of this friend. This approach is ecologically valid, as many people search the web on behalf of friends or family members (Fox and Duggan, 2013). This, however, may reflect advice-giving behavior, whereas searching for information for oneself is considered help-seeking behavior (Schulz et al., 2021) and may yield different outcomes. Second, we included a well-known disorder (depression) and a lesser-known disorder (social phobia), and our results showed differences in the effectiveness of internet searches for each. Besides these extremes, it would also be beneficial to include a moderately well-known mental disorder, such as anorexia nervosa. Finally, the MAKS – the scale used to assess mental health knowledge - had low internal consistency. Consequently, mental health knowledge may not have been measured precisely, potentially reducing our ability to detect smaller associations. In sum, these findings should be replicated using a self-directed search, including one or more additional disorders, and with a more reliable measure of mental health knowledge.

## 3 Experiment 2

To address the limitations discussed and replicate our findings, we repeated the experiment with three minor adaptations using an independent sample. First, we instructed participants to imagine experiencing the symptoms described in the vignette and included disorder-specific questionnaires to screen for any symptoms they may be experiencing in real life. Second, we replaced the non-clinical vignette (grief) with one on anorexia nervosa. This change was made, first because the grief vignette did not provide substantial value as a control, and second because anorexia nervosa is a moderately well-known disorder, with higher recognition rates than social phobia but lower than depression (Darby et al., 2012; McNicholas et al., 2016; Reavley and Jorm, 2011a). Its inclusion may provide additional insight into the utility of online searches, particularly for the acquisition of information on moderately well-known mental disorders. Finally, we replaced the MAKS with the Mental Health Literacy Scale (O'Connor and Casey, 2015) to improve measurement accuracy. The hypothesis tested in Experiment 2 were the same as those tested in Experiment 1.

### 3.1 Method

#### 3.1.1 Participants

For Experiment 2, the target sample size was again 251 participants, and we recruited *N* = 252 (Age *M* = 30.78, *SD* = 10.54, 77.8% female, 59.1% with college education). Recruitment methods, as well as inclusion and exclusion criteria, were identical to those used in Experiment 1. Of the 252 participants, 123 were allocated to the internet group, and 129 to the control group. The sample closely resembled the sample in Experiment 1. Demographic data, separated by experimental group, are presented in Table 1.

#### 3.1.2 Procedure and materials

Most of the materials used in Experiment 2 were identical to those in Experiment 1, with the exception that we introduced a new vignette on anorexia nervosa. The answers given to this newly introduced vignette were scored with respect to the German national treatment guidelines for eating disorders (AWMF, 2018) whereas the answers to the depression and social phobia vignettes were scored as in Experiment 1. Furthermore, we replaced the Mental Health Knowledge Scale (MAKS; Evans-Lacko et al., 2010) with the Mental Health Literacy Scale (MHLS; O'Connor and Casey, 2015) and added established questionnaires to assess whether participants were also experiencing the symptoms described in the vignette in real life. The procedure was identical to experiment 1.

##### 3.1.2.1 Depression

For participants allocated to the depression vignette, we used the German version of the Beck Depression Inventory-II (BDI-II; Hautzinger et al., 2006) o screen for current depressive symptoms. The BDI-II is a widely used and well-established tool for measuring depressive symptoms at both clinical and subclinical levels. It contains 21 items and demonstrates excellent internal consistency (Kühner et al., 2007). Internal consistency in the present sample was excellent (Cronbach's α = 0.94).

##### 3.1.2.2 Social phobia

To screen for symptoms of social phobia in participants allocated to the social phobia vignette, we used the Social Interaction Anxiety Scale (Mattick and Clarke, 1998). This scale assesses anxiety responses in social interactions (both in groups and dyads) across 20 items on a 5-point Likert scale (1 = “not at all” to 5 = “extremely”). It has demonstrated excellent internal consistency in previous studies (Cronbach's α = 0.86–0.93) (Hedman et al., 2010), and internal consistency was also excellent in the present sample (Cronbach's α = 0.91).

##### 3.1.2.3 Anorexia nervosa

For participants allocated to the anorexia nervosa vignette, we used the Anorexia Nervosa Inventory for Self-Rating (ANIS; Fichter and Keeser, 1980) to assess symptoms of anorexia nervosa. The ANIS includes 31 items, assessed on a 6-point Likert scale (1 = “not at all” to 6 = “very much”), across six factors: Consciousness, Feelings of Insufficiency, Anancasm, Adverse Effects of Meals, Sexual Anxieties, and Bulimia. Internal consistency ranges from Cronbach's α = 0.89–0.94, depending on the sample in previous studies and was slightly lower, but still good, in our sample (Cronbach α = 0.87).

##### 3.1.2.4 Mental health knowledge

The Mental Health Literacy Scale (MHLS; O'Connor and Casey, 2015) was used to assess mental health knowledge on a 4-point Likert scale (1 = very unlikely, 4 = very likely). The MHLS previously demonstrated good internal consistency (Cronbach's α = 0.87) and internal consistency was acceptable in the present sample (Cronbach's α = 0.79). For the purposes of this experiment, we only used items 1–15, as these items specifically assess mental health knowledge. Items 16–35 measure other facets of mental health literacy, such as attitudes toward mental disorders.

#### 3.1.3 Data analysis

Data analysis was identical to Experiment 1, with the exception that the experience of the symptoms described in the vignette, measured with the respective questionnaire (BDI-II, SIAS, or ANIS) was added as a covariate in both ANCOVAs.

### 3.2 Results

#### 3.2.1 Descriptives: digital health information use

Of the entire sample, 0.4% very often (i.e., daily), 2.4% often, 10.3% sometimes, 12.3% rarely, and 74.6% never used mental health apps. Again, searching the internet for mental health information was more common: 6.7% used it very often, 19.8% often, 28.2% sometimes, 29.8% rarely, and 15.5% never. In addition, 89.7% reported that they would consider online mental health information when making health-related decisions.

#### 3.2.2 Accuracy hypothesis

A 2 (group: control vs. internet) × 3 (vignette: depression vs. social phobia vs. grief) ANCOVA was calculated with percentage of accurate answers as the dependent variable and the covariates symptom experience, mental health knowledge, eHealth literacy, use of mental health apps, and use of online mental health information. Results showed a significant interaction effect for group x disorder, *F*(2, 241) = 10.64 *p* < 0.001, partial *η*^*2*^ = 0.081, as well as significant main effects for group, *F*(1, 241) = 8.15, *p* = 0.005, partial *η*^*2*^ = 0.033, but a significant main effect for disorder, *F*(2, 241) = 8.66, *p* < 0.001, partial *η*^*2*^ = 0.067. Symptom experience, use of mental health apps and use of online mental health information, and subjectively measured eHealth literacy were not significant covariates, (Symptom Experience: *F*(1, 241) = 0.34, *p* = 0.559, partial *η*^*2*^ = 0.001; Use of mental health apps: *F*(1, 241) = 0.93, *p* = 0.336, partial *η*^*2*^ = 0.004; Use of online mental health information: *F*(1, 241) = 0.03, *p* = 0.864, partial *η*^*2*^ = 0.000; eHealth literacy: *F*(1, 241) = 0.01, *p* = 0.900, partial *η*^*2*^ = 0.000), but mental health knowledge was, *F*(1, 241) = 4.82, *p* = 0.029, partial *η*^*2*^ = 0.020.

Follow-up tests on the interaction effect (significance threshold of α = 0.0167 [0.05/3]) showed that for social phobia, the internet group (*M* = 84.01, *SD* = 20.74) provided more correct answers than the control group (*M* = 60.54, *SD* = 23.36), *t*(82) = 4.87, *p* < 0.001, *Cohen's d* = 1.05. For depression, there were no significant differences between the internet group (*M* = 83.08, *SD* = 16.22) and control group (*M* = 80.40, *SD* = 21.60) regarding the percentage of accurate answers, *t*(79) = −0.63 *p* = 0.267, *Cohen's d* = −0.14. Finally, there were no significant differences between the internet group (*M* = 69.10, *SD* = 14.26) and the control group (*M* = 71.75, *SD* = 11.32) for anorexia nervosa, *t*(85) = −096, *p* = 0.170, *Cohen's d* = −0.21. These results suggest that an internet search helped participants give more accurate answers about social phobia, a less well-known disorder, but not about depression or anorexia nervosa.

In the control group, a one-way ANOVA with post hoc Tukey HSD tests was significant, *F*(2, 126) = 11.17, *p* < 0.001, and showed that participants provided significantly fewer correct answers for social phobia compared with depression, MD = 19,85, *p* < 0.001, or anorexia nervosa, MD = 11.01, *p* < 0.001. Further, participants gave fewer correct answers for anorexia nervosa compared with depression, MD = 8.64, *p* < 0.001. In the experimental group, individuals gave a similar percentage of correct answers for depression and social phobia, MD = 0.03, *p* = 0.810, but provided fewer correct answers on anorexia nervosa compared to depression, MD = −13.98, *p* < 0.001, and social phobia, MD = −12.91, *p* < 0.001. See Fig. 1 for an illustration of the effect. The percentage of accurate answers to each question is listed in Table 2.

#### 3.2.3 Decision hypothesis

A 2 (group: control vs. internet) × 3 (vignette: depression vs. social phobia vs. grief) ANCOVA with preparation for decision making as dependent variable and the covariates symptom experience, mental health knowledge, eHealth literacy, use of mental health apps, and use of online mental health information showed no significant interaction effect for group x disorder, *F*(2, 241) = 1.29, *p* = 0.278, partial *η*^*2*^ = 0.011, no significant main effect for group, *F*(1, 241) = 0.41, *p* = 0.524, partial *η2* = 0.002, and no significant main effect for disorder, *F*(2, 241) = 0.13, *p* = 0.883, partial *η2* = 0.001. Furthermore, none of the covariates reached significance, all *F's* ≤ 1.73, all *p's* ≥ 0.189, partial *η*^*2*^*'s* ≤ 0.007. This shows that searching the web did not lead participants to feel more prepared for a health decision overall, and that being personally affected by a disorder did not influence how prepared individuals felt after a web search. Furthermore, the vignette participants were shown made no difference.

## 4 Discussion

### 4.1 General discussion

In theory, the internet offers a valuable means for individuals to access mental health information and prepare for health decision-making; however, its effectiveness in practice remains a subject of debate. We assessed whether a web search helps individuals find accurate information about mental disorders and increases their preparedness for health decision-making in two online experiments. Importantly, since the second experiment is a conceptual replication of the first, and the findings are consistent across both experiments, we consider the validity and robustness of our results to be particularly strong. Our results showed that individuals gain new insights about social phobia – a lesser-known disorder – through an internet search, whereas this is not the case for depression or anorexia nervosa – comparatively better-known mental disorders. This suggests that online searches primarily improve knowledge of lesser-known disorders (Tay et al., 2018), which partially supports our accuracy hypothesis. Regardless of whether an internet search led to an information gain, results from both experiments demonstrate that it does not increase individuals' sense of preparedness to make a related health decision. This finding contradicts our decision hypothesis and calls for a closer examination of its implications.

To our knowledge, these are the first studies to experimentally assess individuals' ability to find mental health information on the internet. Our results align with a systematic review, which shows that online health interventions are particularly effective in increasing knowledge about lesser-known disorders, such as anxiety disorders (Tay et al., 2018). This is likely because the internet offers a wide range of information and can empower individuals to learn about disorders they may otherwise never encounter (Johnston et al., 2013; Sillence et al., 2007). Specifically, an internet search consistently helped individuals identify the correct diagnosis, the most helpful treatments, and appropriate medication in our experiments. Given that many individuals turn to the internet to find information about unfamiliar health problems (Diviani et al., 2015), it is highly beneficial that an internet search can improve knowledge in this domain.

However, our findings also show that internet searches do not lead to information gain when searching for information about better-known mental disorders. In part, this could be due to a ceiling effect: as participants already possessed a foundational understanding of mental disorders such as depression or anorexia nervosa, information found online likely offered little to no added value. Consistent with this interpretation, our control groups provided significantly more accurate answers to questions about depression (80–81%) and anorexia nervosa (71%), than to questions about social phobia (58–60%). In the experimental groups, there were largely no differences in the percentage of accurate answers across different mental disorders (69–84%). Therefore, an internet search likely allowed individuals to catch up on their knowledge of less familiar mental disorders. It is further worth noting that the questions assessed different aspects of mental health knowledge, ranging from the recognition of mental health problems to treatment-related information. Descriptively, treatment-related questions were generally associated with lower accuracy. Consequently, raising knowledge about well-known disorders or supporting treatment-related decisions may require more than the free navigation of a website. Instead, structured and interactive programs tailored to a specific audience may be needed (Bizzotto et al., 2023; Brijnath et al., 2016). One such program that has been shown to be effective and is available to the public is MoodGYM (Christensen et al., 2004).

Interestingly, participants' performance in the internet search task was not associated with their self-reported eHealth literacy. This finding is consistent with recent evidence suggesting that commonly used subjective measures of eHealth literacy, such as the eHEALS, show limited correspondence with objectively assessed eHealth literacy skills (Crocker et al., 2023). Together with recent evidence, these findings support the use of performance-based assessments to complement subjective measures of eHealth literacy.

Individuals did not feel more prepared to make health decisions following an online search for symptom- and treatment-related mental health information, even when they gained novel insights. One possible explanation for this is the generally low and inconsistent quality of online health information (Khazaal et al., 2008, Khazaal et al., 2012; Morel et al., 2008; Zermatten et al., 2010), which may undermine users' confidence in the information they find. Thus, the quality of health information requires significant improvement if the web is to become a trustworthy information resource (Daraz et al., 2019). Awareness of these quality issues has likely increased in recent years, partly due to the COVID-19 pandemic and the widespread dissemination of inadequate health information online (Siebenhaar et al., 2020). Frequent encounters with such poorly curated content may be overwhelming and may make it difficult to process information effectively, often leading to confusion, anxiety (Pollock et al., 2004), heightened risk perceptions (Han et al., 2006), and ultimately to low trust in online health sources (Freeman et al., 2020; Zheng et al., 2023). One possible explanation is that when individuals do not trust the information they find, even newly acquired knowledge may fail to translate into a greater sense of preparedness. However, because trust in online health information was not assessed in the present study, this interpretation remains speculative and should be examined in future research.

Although individuals did not feel better prepared for health decision-making following a web search, approximately 89% reported that they would consider information found on the internet when making health-related decisions. This underscores the internet's role as one of the most important sources of health information, highlighting both its potential and the risks it entails. Different professional groups may draw different conclusions from these findings. Ideally, data scientists and government agencies should intensify their efforts to improve the quality of online health information and take more proactive steps to curb the spread of misinformation. In parallel, psychotherapists and general practitioners could address this challenge by guiding their patients' online searches and discussing the validity of the information they encounter. This approach could help mitigate misinformation and even strengthen the therapeutic relationship (Tan and Goonawardene, 2017). Further, the exchange of information could serve as a foundation for shared decision-making (Dahl et al., 2021; McMullan, 2006). Finally, researchers should examine the content individuals encounter online more closely, address potential biases in online mental health information-seeking behavior, and investigate strategies to counteract such biases.

### 4.2 Strengths and limitations

Although we addressed several limitations in Experiment 2 certain points must still be taken into consideration. First, the samples in both studies were not representative of the general population, as most participants were young, highly educated, and female. This limits the generalizability of our findings, particularly since previous studies suggest that young females are among the most frequent users of online health information (Rice, 2006; Tennant et al., 2015). However, neither gender nor age were associated with successful search outcomes. The percentage of individuals who had previously used the internet as a mental health information resource was also much higher in our studies (80–85%) compared to earlier studies with more diverse samples (e.g., 26–52%; Bach and Wenz, 2020; Deutschland-Barometer Depression, 2025; Freytag et al., 2023; Siebenhaar et al., 2020). This may be due to the general increase in internet use, or our sample may have been particularly experienced in dealing with online health information. Either way, the present findings may not generalize to populations with lower eHealth literacy or less experience using online health information. Future research should, therefore, examine whether the observed effects replicate in more diverse, less internet experiences groups.

Furthermore, we conducted both studies online, which made the study environment less controllable (Clifford and Jerit, 2014). Therefore, we cannot exclude the possibility that participants in the control group also searched the web to answer questions. However, we consider this highly unlikely because we specifically instructed participants not to and because the percentages of accurate answers were similar for both experiments. This makes us confident that conducting our experiments online allowed us to collect a larger sample without compromising the quality of our data.

### 4.3 Conclusion

Our results show that an internet search can promote an increase in knowledge about social phobia and possibly also about other less well-known mental disorders. This represents a clear advantage of searching for mental health information online. However, an internet search does not appear to expand knowledge about mental disorders with which individuals are already relatively familiar. Regardless of a potential information gain, individuals did not feel better prepared for health decision making after searching the internet. These findings indicate that the utility of a web search is limited. To effectively improve mental health knowledge – particularly regarding more commonly known disorders – more specialized, structured, and interactive interventions may be needed.

## CRediT authorship contribution statement

**Katharina U. Siebenhaar:** Conceptualization, Methodology, Formal analysis, Investigation, Data curation, Writing – original draft, Writing – review & editing, Visualization, Project administration. **Georg W. Alpers:** Conceptualization, Resources, Writing – review & editing, Supervision, Funding acquisition.

## Declaration of Generative AI and AI-assisted technologies in the writing process

The authors declare the use of OpenAI's ChatGPT (2025 Version) for proofreading and minor language editing. The tool was not used for other tasks. Responsibility for the final manuscript lies entirely with the authors.

## Funding

This project was funded by the State of Baden-Württemberg as part of the research consortium “digilog@bw” (Digitalization in dialogue). KUS received additional stipends from the University of Mannheim's Graduate School of Economic and Social Sciences and from the Funding Program for the Promotion of Postgraduates of the Land of Baden-Württemberg (LGF). GWA received additional funding from the 10.13039/501100002347Federal Ministry for Education and Research (Bundesministeriums für Bildung und Forschung, BMBF) grant number 03IHS264B (TransforMA).

## Declaration of competing interest

The authors declare that they have no known competing financial interests or personal relationships that could have appeared to influence the work reported in this paper.
