# Supplementary material for: Consulting Dr. Google: Two experimental studies on seeking mental health information online
Source: Internet Interv. 2026 Aug 21;46:100992. doi: 10.1016/j.invent.2026.100992 (PMC13659325; doi:10.1016/j.invent.2026.100992)
Supplement: Supplementary file 1 [file mmc1.docx]

**Supplementary Material:**

**Consulting Dr. Google: Two Experimental Studies on Seeking Mental Health Information Online**

1. **Materials: Vignettes**
   1.1. **Vignettes Experiment 1 (German)**

Depression (männlich)

**Stellen Sie sich vor, ein sehr guter Freund erzählt Ihnen, dass er sich seit mehreren Wochen niedergeschlagen fühlt und die Freude an vielen Dingen verloren hat. Zudem schläft er sehr schlecht, ist ständig müde, hat wenig Appetit und große Schwierigkeiten, sich zu konzentrieren. In den letzten Wochen hat Ihr Freund auch öfter geschildert, dass er sich innerlich leer fühlt und nicht weiß, wie jemals wieder alles besser werden könnte. Da ihm im Alltag alles zu viel wurde, hat er sich gestern von seinem Arzt vorerst krankschreiben lassen.**

**Instruktion Kontrollgruppe: Bitte beantworten Sie die folgenden Fragen. Recherchieren sie die Antworten nicht im Internet.**

**Instruktion Experimentalgruppe: Sie möchten Ihrem Freund gerne helfen und beschließen, im Internet nach Informationen zu recherchieren. Bitte beantworten Sie mithilfe einer Internetrecherche die folgenden Fragen. Beziehen Sie Ihre Antworten dabei nicht ausschließlich auf Ihre persönliche Einstellung oder Ihr aktuelles Wissen, sondern geben Sie die Ihrer Meinung nach bestmögliche Antwort auf Basis Ihrer Recherche.**

**Depression (weiblich)**

Stellen Sie sich vor, eine sehr gute Freundin erzählt Ihnen, dass sie sich seit mehreren Wochen niedergeschlagen fühlt und die Freude an vielen Dingen verloren hat. Zudem schläft sie sehr schlecht, ist ständig müde, hat wenig Appetit und große Schwierigkeiten, sich zu konzentrieren. In den letzten Wochen hat Ihre Freundin auch öfter geschildert, dass sie sich innerlich leer fühlt und nicht weiß, wie jemals wieder alles besser werden könnte. Da ihr im Alltag alles zu viel wurde, hat sie sich gestern von ihrer Ärztin vorerst krankschreiben lassen.

**Instruktion Kontrollgruppe: Bitte beantworten Sie die folgenden Fragen. Recherchieren sie die Antworten nicht im Internet.**

**Instruktion Experimentalgruppe:** Sie möchten Ihrer Freundin gerne helfen und beschließen, im Internet nach Informationen zu recherchieren. Bitte beantworten Sie mithilfe einer Internetrecherche die folgenden Fragen. Beziehen Sie Ihre Antworten dabei nicht ausschließlich auf Ihre persönliche Einstellung oder Ihr aktuelles Wissen, sondern geben Sie die Ihrer Meinung nach bestmögliche Antwort auf Basis Ihrer Recherche.

Soziale Phobie (männlich)

Stellen Sie sich vor, ein sehr guter Freund erzählt Ihnen, dass er auf der Arbeit versetzt wurde und nun mehrmals am Tag Kundenkontakt hat. Das bereitet Ihrem Freund große Schwierigkeiten, da er bei Begegnungen mit fremden Menschen stets sehr angespannt ist, keinen Augenkontakt halten kann, stark schwitzt und errötet. Manchmal zittert dabei auch seine Stimme, was die Situation zusätzlich verschlimmert, da er dann umso mehr befürchtet, für unfähig gehalten zu werden. All das belastet Ihren Freund so sehr, dass er seinen Arbeitsalltag nur mit großer Anstrengung bewältigen kann.

**Instruktion Kontrollgruppe: Bitte beantworten Sie die folgenden Fragen. Recherchieren sie die Antworten nicht im Internet.**

**Instruktion Experimentalgruppe:** Sie möchten Ihrem Freund gerne helfen und beschließen, im Internet nach Informationen zu recherchieren. Bitte beantworten Sie mithilfe einer Internetrecherche die folgenden Fragen. Beziehen Sie Ihre Antworten dabei nicht ausschließlich auf Ihre persönliche Einstellung oder Ihr aktuelles Wissen, sondern geben Sie die Ihrer Meinung nach bestmögliche Antwort auf Basis Ihrer Recherche.

Soziale Phobie (weiblich)

Stellen Sie sich vor, eine sehr gute Freundin erzählt Ihnen, dass sie auf der Arbeit versetzt wurde und nun mehrmals am Tag Kundenkontakt hat. Das bereitet Ihrer Freundin große Schwierigkeiten, da sie bei Begegnungen mit fremden Menschen stets sehr angespannt ist, keinen Augenkontakt halten kann, stark schwitzt und errötet. Manchmal zittert dabei auch ihre Stimme, was die Situation zusätzlich verschlimmert, da sie dann umso mehr befürchtet, für unfähig gehalten zu werden. All das belastet Ihre Freundin so sehr, dass sie ihren Arbeitsalltag nur mit großer Anstrengung bewältigen kann.

**Instruktion Kontrollgruppe: Bitte beantworten Sie die folgenden Fragen. Recherchieren sie die Antworten nicht im Internet.**

**Instruktion Experimentalgruppe:** Sie möchten Ihrer Freundin gerne helfen und beschließen, im Internet nach Informationen zu recherchieren. Bitte beantworten Sie mithilfe einer Internetrecherche die folgenden Fragen. Beziehen Sie Ihre Antworten dabei nicht ausschließlich auf Ihre persönliche Einstellung oder Ihr aktuelles Wissen, sondern geben Sie die Ihrer Meinung nach bestmögliche Antwort auf Basis Ihrer Recherche.

Trauer (männlich)

Stellen Sie sich vor, ein sehr guter Freund erzählt Ihnen, dass er gestern Morgen erfahren hat, dass sein Großvater gestorben ist. Er hatte seinen Großvater in den letzten Jahren nur selten gesehen, da dieser mehr als 600 Kilometer entfernt wohnte. Dennoch ist er über dessen Tod bestürzt und sehr traurig. Nachdem er die Nachricht erhalten hatte, konnte er nicht zur Arbeit gehen. Stattdessen lag er den ganzen Tag im Bett und schaute sich alte Fotoalben an. Heute war er wieder arbeiten, musste aber am Abend beim Telefonat mit seinen Eltern erneut weinen.

**Instruktion Kontrollgruppe: Bitte beantworten Sie die folgenden Fragen. Recherchieren sie die Antworten nicht im Internet.**

**Instruktion Experimentalgruppe:** Sie möchten Ihrem Freund gerne helfen und beschließen, im Internet nach Informationen zu recherchieren. Bitte beantworten Sie die folgenden Fragen mithilfe einer Internetrecherche. Beziehen Sie Ihre Antworten dabei nicht ausschließlich auf Ihre persönliche Meinung oder Ihr aktuelles Wissen, sondern geben Sie die Ihrer Einschätzung nach bestmögliche Antwort auf Grundlage Ihrer Recherche.

Trauer (weiblich)

Stellen Sie sich vor, eine sehr gute Freundin erzählt Ihnen, dass sie gestern Morgen erfahren hat, dass ihre Großmutter gestorben ist. Sie hatte ihre Großmutter in den letzten Jahren nur selten gesehen, da diese mehr als 600 Kilometer entfernt wohnte. Dennoch ist sie über deren Tod bestürzt und sehr traurig. Nachdem sie die Nachricht erhalten hatte, konnte sie nicht zur Arbeit gehen. Stattdessen lag sie den ganzen Tag im Bett und schaute sich alte Fotoalben an. Heute war sie wieder arbeiten, musste aber am Abend beim Telefonat mit ihren Eltern erneut weinen.

**Instruktion Kontrollgruppe: Bitte beantworten Sie die folgenden Fragen. Recherchieren sie die Antworten nicht im Internet.**

**Instruktion Experimentalgruppe:** Sie möchten Ihrer Freundin gerne helfen und beschließen, im Internet nach Informationen zu recherchieren. Bitte beantworten Sie die folgenden Fragen mithilfe einer Internetrecherche. Beziehen Sie Ihre Antworten dabei nicht ausschließlich auf Ihre persönliche Meinung oder Ihr aktuelles Wissen, sondern geben Sie die Ihrer Einschätzung nach bestmöglicher Antwort auf Grundlage Ihrer Recherche.

**1.2 Vignettes Experiment 1 (English)**

Depression (male)

Imagine a very close male friend tells you that he has been feeling down for several weeks and has lost interest in many things he used to enjoy. He also sleeps poorly, is constantly tired, has little appetite, and finds it very difficult to concentrate. In recent weeks, your friend has also shared that he feels emotionally empty and cannot imagine how things could ever get better. Because everyday life has become too overwhelming, he was signed off work by his doctor yesterday.

Control group: Please answer the following questions. Do not research the answers on the internet.

Experimental group: You want to help your friend and decide to search the internet for information. Please answer the following questions using an internet search. Do not base your responses solely on your opinion or current knowledge but provide what you believe to be the best possible answer based on your internet search.

Depression (female)

Imagine a very close female friend tells you that she has been feeling down for several weeks and has lost interest in many things she used to enjoy. She also sleeps poorly, is constantly tired, has little appetite, and finds it very difficult to concentrate. In recent weeks, your friend has also shared that she feels emotionally empty and cannot imagine how things could ever get better. Because everyday life has become too overwhelming, she was signed off work by her doctor yesterday.

Control group: Please answer the following questions. Do not research the answers on the internet.

Experimental group: You want to help your friend and decide to search the internet for information. Please answer the following questions using an internet search. Do not base your responses solely on your opinion or current knowledge but provide what you believe to be the best possible answer based on your internet search.

Social Phobia (male)

Imagine a very close male friend tells you that he has been reassigned at work and now has to interact with customers several times a day. This is causing him great distress, as he feels extremely tense in contact with strangers, cannot maintain eye contact, and tends to sweat and blush heavily. Sometimes his voice even begins to tremble, which only makes things worse, because he then fears even more that others will see him as incompetent. This situation is so burdensome that your friend can only get through the workday with great effort.

Control group: Please answer the following questions. Do not research the answers on the internet.

Experimental group: You want to help your friend and decide to search the internet for information. Please answer the following questions using an internet search. Do not base your responses solely on your opinion or current knowledge but provide what you believe to be the best possible answer based on your internet search.

Social Phobia (female)

Imagine a very close female friend tells you that she has been reassigned at work and now has to interact with customers several times a day. This is causing her great distress, as she feels extremely tense in contact with strangers, cannot maintain eye contact, and tends to sweat and blush heavily. Sometimes her voice even begins to tremble, which only makes things worse, because she then fears even more that others will see her as incompetent. This situation is so burdensome that your friend can only get through the workday with great effort.

Control group: Please answer the following questions. Do not research the answers on the internet.

Experimental group: You want to help your friend and decide to search the internet for information. Please answer the following questions using an internet search. Do not base your responses solely on your opinion or current knowledge but provide what you believe to be the best possible answer based on your internet search.

Grief (male)

Imagine a very close male friend tells you that he learned yesterday morning that his grandfather has passed away. He had only seen his grandfather rarely in recent years, as he lived more than 600 kilometers away. Still, he is deeply saddened and affected by the death. After receiving the news, he was unable to go to work. Instead, he spent the day in bed looking through old photo albums. Today he returned to work, but during an evening phone call with his parents, he began to cry again.

Control group: Please answer the following questions. Do not research the answers on the internet.

Experimental group: You want to help your friend and decide to search the internet for information. Please answer the following questions using an internet search. Do not base your responses solely on your opinion or current knowledge but provide what you believe to be the best possible answer based on your internet search.

Grief (female)

Imagine a very close male friend tells you that he learned yesterday morning that his grandfather has passed away. He had only seen his grandfather rarely in recent years, as he lived more than 600 kilometers away. Still, he is deeply saddened and affected by the death. After receiving the news, he was unable to go to work. Instead, he spent the day in bed looking through old photo albums. Today he returned to work, but during an evening phone call with his parents, he began to cry again.

Control group: Please answer the following questions. Do not research the answers on the internet.

Experimental group: You want to help your friend and decide to search the internet for information. Please answer the following questions using an internet search. Do not base your responses solely on your opinion or current knowledge but provide what you believe to be the best possible answer based on your internet search.

**1.3 Vignettes Experiment 2 (German)**

Depression

Stellen Sie sich vor, dass Sie sich seit mehreren Wochen niedergeschlagen fühlen und die Freude an vielen Dingen verloren haben. Zudem schlafen Sie sehr schlecht, sind ständig müde, haben wenig Appetit und große Schwierigkeiten, sich zu konzentrieren. In den letzten Wochen hatten Sie häufig das Gefühl, innerlich leer zu sein und nicht zu wissen, wie jemals wieder alles besser werden könnte. Da Ihnen auch der Alltag zunehmend zu viel wurde, haben Sie sich gestern von Ihrem Arzt vorerst krankschreiben lassen.

**Instruktion Kontrollgruppe: Bitte beantworten Sie die folgenden Fragen. Recherchieren sie die Antworten nicht im Internet.**

Instruktion Experimentalgruppe: Sie möchten sich über diese Symptome informieren und beschließen, im Internet zu recherchieren. Bitte beantworten Sie mithilfe einer Internetrecherche die folgenden Fragen. Beziehen Sie Ihre Antworten dabei nicht ausschließlich auf Ihre persönliche Einstellung oder Ihr aktuelles Wissen, sondern geben Sie die Ihrer Einschätzung nach bestmögliche Antwort auf Grundlage Ihrer Recherche.

Soziale Phobie:

Stellen Sie sich vor, dass Sie auf der Arbeit versetzt wurden und nun mehrmals am Tag Kundenkontakt haben. Das bereitet Ihnen große Schwierigkeiten, da Sie bei Kontakt mit fremden Menschen stets sehr angespannt sind, keinen Augenkontakt halten können, stark schwitzen und erröten. Manchmal zittert dann auch Ihre Stimme, was alles noch schlimmer macht, da Sie dann erst recht befürchten für unfähig gehalten zu werden. Das alles belastet Sie so sehr, dass Sie nur unter großer Anstrengung Ihren Arbeitsalltag bewältigen können.

**Instruktion Kontrollgruppe: Bitte beantworten Sie die folgenden Fragen. Recherchieren sie die Antworten nicht im Internet.**

Instruktion Experimentalgruppe: Sie möchten sich über diese Symptome informieren und beschließen, im Internet zu recherchieren. Bitte beantworten Sie mithilfe einer Internetrecherche die folgenden Fragen. Beziehen Sie Ihre Antworten dabei nicht ausschließlich auf Ihre persönliche Einstellung oder Ihr aktuelles Wissen, sondern geben Sie die Ihrer Einschätzung nach bestmögliche Antwort auf Grundlage Ihrer Recherche.

Anorexia Nervosa

Stellen Sie sich vor, dass Sie schon länger Diät halten und mittlerweile sehr schlank sind (170 cm groß, 46 kg, 15.9 BMI), aber dennoch extreme Angst davor haben, zu dick zu sein oder wieder zuzunehmen. Insbesondere Ihren Bauch und Ihre Oberschenkel finden Sie weiterhin sehr dick und verstehen nicht, warum Andere sagen, dass Sie zu dünn sind. Deshalb essen Sie aktuell nur kleine Portionen an Gemüse oder Obst und vermeiden es kalorienreiche Lebensmittel zu essen. Auch treiben Sie sehr viel Sport, um Kalorien zu verbrennen.

**Instruktion Kontrollgruppe: Bitte beantworten Sie die folgenden Fragen. Recherchieren sie die Antworten nicht im Internet.**

Instruktion Experimentalgruppe: Sie möchten sich über diese Symptome informieren und beschließen, im Internet zu recherchieren. Bitte beantworten Sie mithilfe einer Internetrecherche die folgenden Fragen. Beziehen Sie Ihre Antworten dabei nicht ausschließlich auf Ihre persönliche Einstellung oder Ihr aktuelles Wissen, sondern geben Sie die Ihrer Einschätzung nach bestmögliche Antwort auf Grundlage Ihrer Recherche.

**1.4 Vignettes Experiment 2 (English)**

Depression

Imagine that you have been feeling down for several weeks and have lost interest in many things. Additionally, you sleep very poorly, are constantly tired, have little appetite, and have great difficulty concentrating. In recent weeks, you have often felt empty inside and did not know how things could ever get better again. Because everyday life has also become increasingly overwhelming, you asked your doctor yesterday to write you off sick for the time being.

**Control group:** Please answer the following questions. Do not research the answers on the internet.

Experimental group: You would like to inform yourself about these symptoms and decide to do an internet search. Please answer the following questions using online research. Do not base your answers solely on your personal opinion or current knowledge but provide what you consider to be the best possible answer based on your research.

Social Phobia

Imagine that you were transferred at work and now have to deal with customers several times a day. This causes you great difficulty because you are always very tense when interacting with strangers, cannot maintain eye contact, sweat heavily, and blush. Sometimes your voice also trembles, which makes everything worse because you then fear even more that others will think you are incompetent. This is so distressing that you can only get through your workday with great effort.

**Control group:** Please answer the following questions. Do not research the answers on the internet.

Experimental group: You would like to inform yourself about these symptoms and decide to do an internet search. Please answer the following questions using online research. Do not base your answers solely on your personal opinion or current knowledge but provide what you consider to be the best possible answer based on your research.

Anorexia Nervosa

Imagine that you have been dieting for a while and are now very slim (170 cm tall, 46 kg, BMI 15.9), but still have an extreme fear of being fat or gaining weight again. In particular, you continue to see your stomach and thighs as very fat and do not understand why others say you are too thin. Therefore, you are currently only eating small portions of vegetables or fruit and avoid eating calorie-rich foods. You also exercise a lot to burn calories.

**Control group:** Please answer the following questions. Do not research the answers on the internet.

Experimental group: You would like to inform yourself about these symptoms and decide to do an internet search. Please answer the following questions using online research. Do not base your answers solely on your personal opinion or current knowledge but provide what you consider to be the best possible answer based on your research.

2. **Materials:**
   2.1. **Self-Generated Questions**
        2.1.1. **German**
1. Benutzen Sie Apps für psychische Gesundheit?
2. Suchen Sie bei Fragen zu psychischen Beschwerden im Internet nach Informationen?
3. Würden Sie im Internet recherchierte Informationen bei Entscheidungen im Rahmen der Behandlung psychischer Beschwerden berücksichtigen (z.B. während einer Psychotherapie)?

Skala: Nie - selten - manchmal - oft - immer

2.1.2. **English**

4. Do you use mental health apps?
5. Do you search the internet for information about mental health issues?
6. Would you consider information you found online when making decisions related to the treatment of mental health issues (e.g., during psychotherapy)?

Scale: Never – Rarely – Sometimes – Often – Always

2.2. **Questions Regarding the Internet Search**
     2.2.1. **German**

1. Handelt es sich bei den Symptomen um eine Erkrankung (körperlich, psychisch)?
2. **Wie lautet die Diagnose der beschriebenen Erkrankung?**
3. **Sollte Ihre Freundin/ Ihr Freund/ Sollten Sie professionelle Hilfe in Anspruch nehmen?**
4. **An wen sollte Ihr Freund/ Ihre Freundin/ An wen sollten Sie sich wenden?**
5. Was halten Sie für die hilfreichste Behandlung?
6. Falls Ihr Freund/ Ihre Freundin/ Falls Sie Medikamente einnehmen, welche sollten Sie nehmen?
7. Sollte Ihr Freund/ Ihre Freundin/ Sollten Sie mit dieser Symptomatik in eine stationäre Klinik aufgenommen werden?
   2.2.2. **English**
1. Are the symptoms indicative of an illness (physical or psychological)?
2. What is the diagnosis of the described condition?
3. Should your friend / should you seek professional help?
4. Whom should your friend / should you contact?
5. What do you consider the most helpful treatment?
6. If your friend / if you take medication, which one should it be?
7. Should your friend / should you be admitted to an inpatient clinic for these symptoms?
3. **Answers**

| **Depression** | | | |
| --- | --- | --- | --- |
|  | **Question** | Correct Answer | Question Format |
|  | Are the symptoms indicative of an illness (physical or psychological)? | Yes | Multiple Choice |
|  | What is the diagnosis of the described condition? | Depression, Major Depression Depressive Episode, F 32, | Open End |
|  | Should your friend / should you seek professional help? | Yes | Multiple Choice |
|  | Whom should your friend / should you contact? | General Practitioner, Psychiatrist, Psychotherapist | Multiple Choice (more than one correct answer) |
|  | What do you consider the most helpful treatment? | Psychotherapy, Medication, A combination of psychotherapy and medication | Multiple Choice (more than one correct anwer) |
|  | If your friend / if you take medication, which one should it be? | SSRI | Multiple Choice |
|  | Should your friend / should you be admitted to an inpatient clinic for these symptoms? | No | Multiple Choice |

| **Social Phobia** | | | |
| --- | --- | --- | --- |
|  | **Question** | Correct Answer | Question Format |
|  | Are the symptoms indicative of an illness (physical or psychological)? | Yes | Multiple Choice |
|  | What is the diagnosis of the described condition? | Social Phobia, Social Anxiety Disorder, F40.1 | Open End |
|  | Should your friend / should you seek professional help? | Yes | Multiple Choice |
|  | Whom should your friend / should you contact? | General Practitioner, Psychiatrist, Psychotherapist | Multiple Choice (more than one correct answer) |
|  | What do you consider the most helpful treatment? | Psychotherapy, Psychotherapy and Medication | Multiple Choice (more than one correct anwer) |
|  | If your friend / if you take medication, which one should it be? | SSRI | Multiple Choice |
|  | Should your friend / should you be admitted to an inpatient clinic for these symptoms? | No | Multiple Choice |

| **Grief** | | | |
| --- | --- | --- | --- |
|  | **Question** | Correct Answer | Question Format |
|  | Are the symptoms indicative of an illness (physical or psychological)? | No | Multiple Choice |
|  | What is the diagnosis of the described condition? | No Disorder, / | Open End |
|  | Should your friend / should you seek professional help? | No | Multiple Choice |
|  | Whom should your friend / should you contact? | friends and family | Multiple Choice (more than one correct answer) |
|  | What do you consider the most helpful treatment? | Nothing (no treatment, wait and see) | Multiple Choice (more than one correct anwer) |
|  | If your friend / if you take medication, which one should it be? | No medication | Multiple Choice |
|  | Should your friend / should you be admitted to an inpatient clinic for these symptoms? | No | Multiple Choice |

| **Anorexia Nervosa** | | | |
| --- | --- | --- | --- |
|  | **Question** | Correct Answer | Question Format |
|  | Are the symptoms indicative of an illness (physical or psychological)? | Yes | Multiple Choice |
|  | What is the diagnosis of the described condition? | Anorexia Nervosa, Anorexia, F50 | Open End |
|  | Should your friend / should you seek professional help? | Yes | Multiple Choice |
|  | Whom should your friend / should you contact? | General Practitioner, Psychiatrist, Psychotherapist | Multiple Choice (more than one correct answer) |
|  | What do you consider the most helpful treatment? | Psychotherapy | Multiple Choice (more than one correct anwer) |
|  | If your friend / if you take medication, which one should it be? | No Medication | Multiple Choice |
|  | Should your friend / should you be admitted to an inpatient clinic for these symptoms? | No | Multiple Choice |
